# Variation in hatching responses of *Nematodirus battus* eggs to temperature experiences

**DOI:** 10.1186/s13071-020-04368-9

**Published:** 2020-09-29

**Authors:** Lynsey A. Melville, Jan Van Dijk, Sian Mitchell, Giles Innocent, David J. Bartley

**Affiliations:** 1 Disease Control, Moredun Research Institute, Pentlands Science Park, Bush Loan, Penicuik, EH26 0PZ UK; 2 Zoetis, Birchwood Building, Springfield Drive, Leatherhead, KT22 7LP UK; 3 Animal and Plant Health Agency, Job’s Well Road, Johnstown, Carmarthen, SA31 3EZ UK; 4 Biomathematics and Statistics Scotland, JCMB, King’s Buildings, Peter Guthrie Tait Road, Edinburgh, EH9 3FD UK

**Keywords:** *Nematodirus battus*, Hatching, Chill stimulus, Climate change, Seasonality, Nematode

## Abstract

**Background:**

*Nematodirus battus*, unlike most other gastrointestinal nematodes, undergoes maturation to an infective larva within the egg. Historically, eggs were considered to require a period of chilling over winter followed by a period of temperature above 10 °C for synchronous hatching to occur (generally in spring). Anecdotal reports of *Nematodirus* infection out-with spring in veterinary journals and the farming press suggest that the concentrated pasture abundance of *N. battus* infective larvae may be changing. In order for control practices to be adapted, and unexpected disease outbreaks to be avoided, it is important to quantify how parasite epidemiology is changing and research the drivers behind it.

**Method:**

The present study investigated the *in vitro* hatching response to temperature experiences (with and without a period of chilling) for egg samples of 90 *N. battus* populations obtained from 73 commercial sheep farms. Six aliquots of larvated eggs were prepared per population, three aliquots were placed at 4 °C for 6  weeks to provide a chill stimulus then incubated at the optimal hatching temperature for the species. The remaining three aliquots of eggs were incubated at the hatching temperature without a prior chill stimulus and the number of hatched larvae was compared between treatments.

**Results:**

Median hatch rate across all populations with chilling was 45% (95% CI: 42–48%) and without chilling was 4% (95% CI: 2–6%). Inter-population variation in hatching ranged from 0 to 87% of eggs able to hatch in the absence of a chill stimulus, mean non-chill hatching was 13 ± 2% of eggs (mean ± SE). Non-chill hatching rates were greater than chilled hatching rates in seven of the 90 populations tested.

**Conclusions:**

Clearly, the variation in hatching responses to temperature experience is very large and therefore the seasonality of the parasite may vary not only between regions but also at farm level. In contrast to what previous work has suggested, there was a geographical trend towards higher non-chill hatching in the Northern parts of the UK.
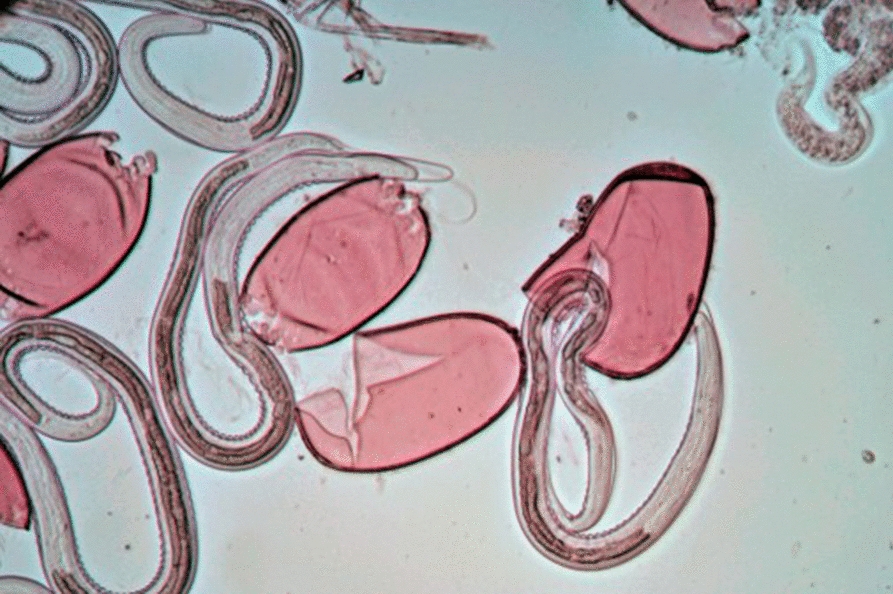

## Background

*Nematodirus battus* is endemic in many parts of northern Europe and represents a significant economic and welfare burden for UK sheep farming [1]. This highly seasonal parasite is typically responsible for acute disease in lambs in late spring [2, 3] causing diarrhoea, production loss and potential death by dehydration following severe hypersensitivity reactions within the small intestine.

*Nematodirus battus* has a direct parasitic life-cycle but, as opposed to most other gastrointestinal nematodes infecting domesticated ruminants, the infective larvae (L_3_) develop within the egg rather than on pasture. Developed L_3_ typically remain encased within the egg throughout the winter months. Once environmental conditions become optimal for larval survival, i.e. mean day and night temperature greater than 10 °C for 10 days [4], synchronous hatching of eggs occurs, resulting in large numbers of infectious larvae on pasture in spring [2]. *Nematodirus battus* is believed to be a species of arctic origin [5], for which increased cold hardiness and longevity on pasture could be important adaptations, increasing the likelihood of survival during periods of host absence. However, given modern, intensive, sheep farming practices where hosts are plentiful year-round, these adaptations may not be as crucial [6].

The winter/chill stimulus was originally believed to be essential to facilitate *N. battus* hatching [4] but more recent work has demonstrated egg populations hatching without chilling [6, 7]. Reliance on a chill stimulus prior to successful hatching of eggs restricted the emergence of *N. battus* larvae to spring, adaptation of a proportion of eggs to hatch in the absence of a chill stimulus (non-chill hatching) could result in infective larvae being present on pasture at different times of year. The prevalence of non-chill hatching throughout the UK has not yet been quantified and the precise role that it plays in the epidemiology and biology of the parasite or the drivers of this phenomenon are unknown. However, it has been proposed that there may be selective pressure on the parasite, applied by climate change and farm management practices designed to control parasite abundance, to respond to hatching opportunities outside of spring [6, 7].

Current *N. battus* control strategies and advice are based largely on the strategic administration of anthelmintics, typically benzimidazole (1-BZ) compounds due to their high safety index for use in young stock and prolonged effectiveness against *Nematodirus* species [8]. The recent emergence of BZ-resistance in UK *N. battus* populations [9–11] appeared to occur around the same time as variable hatching was observed in the species [6, 7], leading to the hypothesis that the two phenomena could be linked. The availability of *N. battus* larvae during summer and autumn could result in greater exposure to BZ treatment, therefore increasing the selection pressure for anthelmintic resistance in the species. Benzimidazole resistance in this parasite is predominantly conferred by a single point mutation at codon 200 of the β-tubulin isotype 1 gene [10, 11].

The timing of anthelmintic administration and management interventions often rely on pre-determined dates, based on infection in previous years, which fails to account for the impact of changing climate on hatching dates of parasite eggs. Forecast models are a useful tool which are becoming more widely used, these predict the hatch date of *N. battus* eggs based on climatic factors however, these are based solely on the traditional expectation of ‘chilled hatching’ of *N. battus* eggs in spring (i.e. eggs require a chill stimulus in order to hatch). Both methods leave the sheep industry vulnerable to unexpected disease at different times of year. Greater knowledge of the prevalence of non-chill hatching (i.e. eggs which do not require a chill stimulus in order to hatch) and possibly the drivers which control this behaviour are therefore key to the development of improved, sustainable control practices.

The aim of the present study was to quantify the hatching responses and associated β-tubulin codon 200 genotypes of a large number of UK *N. battus* populations under two different temperature experiences (with and without a chill stimulus) to explore the variation in hatching behaviour at the individual farm and regional level, with a view to inform on-farm control policies.

## Methods

### Sample collection

**Fig. 1:**
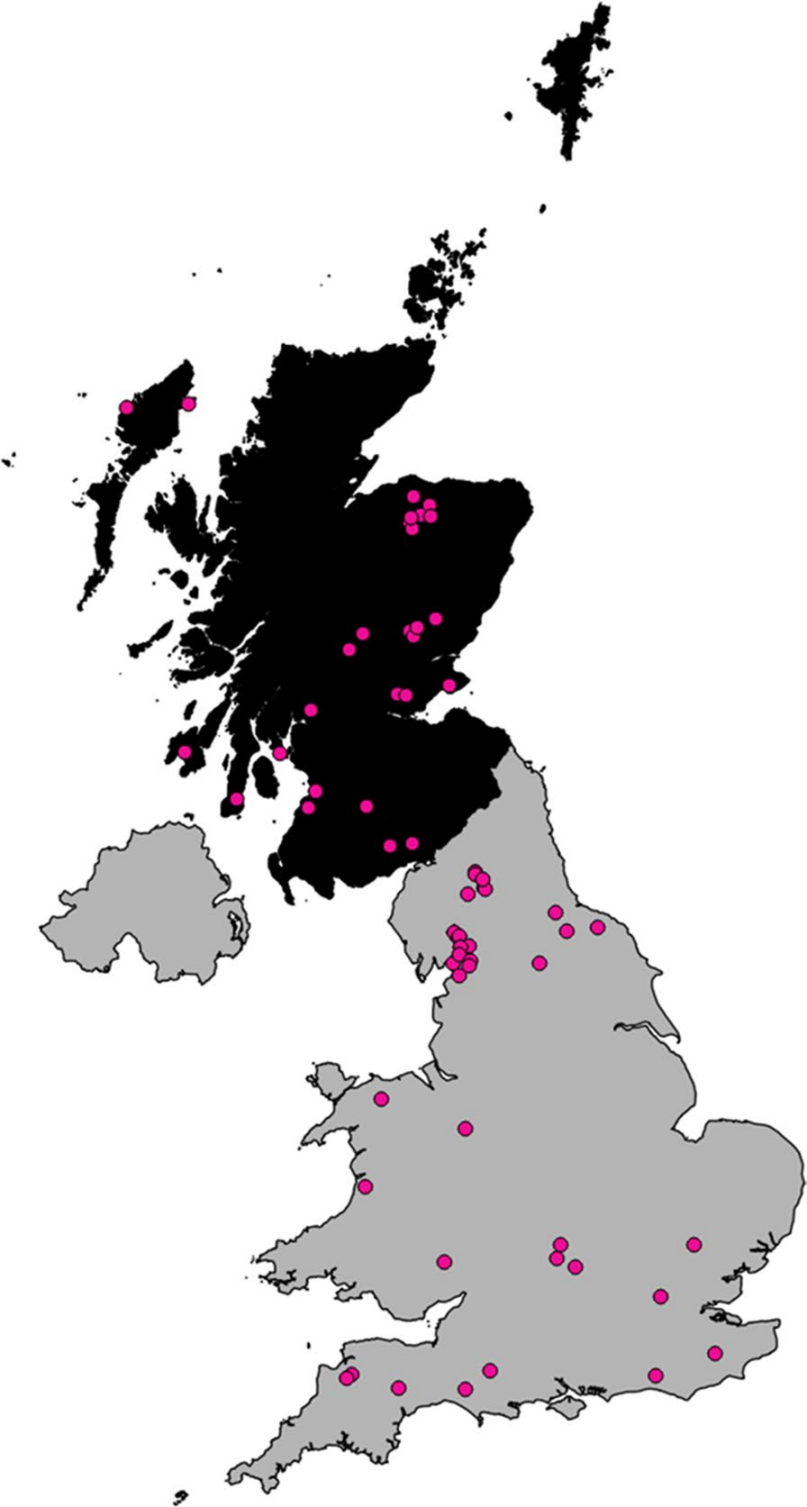
Map of the UK with the origins of *Nematodirus battus* populations included in the hatching study marked

A total of 90 *N. battus* populations from 73 UK commercial farms were collected between June and August in 2015 and 2016 (Fig. 1). Populations originating from the same farm were collected at different time points or from fields with different grazing histories. Sampling was restricted to spring/summer in order to standardise the likely origin of eggs, i.e. these eggs are likely to have originated from adult worms which developed after infection with spring-hatched larvae. A total of 10 populations were submitted by Scotland’s Rural College (SRUC) surveillance centres in Ayr and Dumfries. Samples were collected opportunistically in 2015 in a non-random fashion (*n* = 61). Sampling in 2016 (*n* = 19) was targeted to regions which were under-represented by the *N. battus* sample biobank collected in 2015 but which appeared to have significant sheep density (sheep density data from the Office for National Statistics in 2009 was mapped using QGIS (Las Palmas version 2.18); data source Geo-wiki). Farms in the target regions were contacted *via* local advisors, veterinarians and the Animal and Horticulture Development Board (AHDB).

Farmers were instructed to collect 10 fresh lamb faecal samples from the ground, sealing each in an individual re-sealable bag with excess air removed, samples were then packaged following Royal Mail guidelines for biological samples and posted back to the laboratory at Moredun Research Institute, Midlothian, Scotland. Upon arrival, samples were stored at 4 °C to suspend development prior to processing. Samples which were submitted by SRUC and APHA surveillance centres were packaged in air-tight containers for postage and stored at 4 °C upon arrival, prior to processing. Eggs were stored in faecal matter at 4 °C for between 1 and 33 days (mean 11, median 9). At the point of faecal egg counting, eggs appeared morphologically un-developed; only morulae were observed, and no early-stage larvae within the egg shells. The impact of chilling prior to development was deemed minimal as known physiological changes as a result of chilling are believed to occur within the developed larvae, rather than the undeveloped egg [12].

### Sample preparation

Faecal egg counts were conducted on all samples using the salt floatation method described by Jackson & Christie [13]. *Nematodirus battus* eggs were differentiated from other *Nematodirus* species morphologically and samples containing co-infection with other *Nematodirus* species were excluded from the hatching experiment for ease of interpretation of the results. The average faecal egg count of samples was 178 *N. battus* eggs per gram (EPG, range 6–1250).

**Fig. 2:**
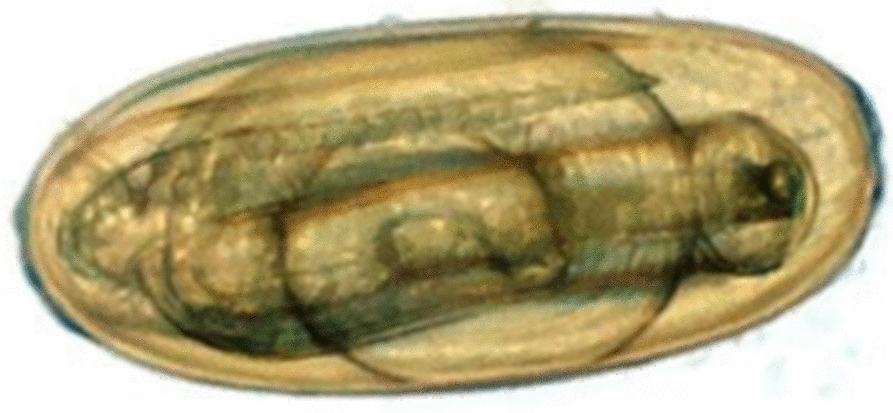
Picture of a *Nematodirus battus* egg at the embryonated stage with third-stage larva visible within the egg shell (a larvated egg)

Eggs were extracted from faeces by differential sieving with *N. battus* eggs collected on a 62 µm sieve, allowing smaller strongyle eggs to pass through. Saturated sodium chloride solution flotation (NaCl; specific gravity 1.2) was used to extract eggs from faecal debris within the filtrate. Eggs were washed with excess tap water to remove remaining NaCl and placed into non-air-tight jars with tap water. Egg cultures were stored at ambient room temperature (~ 20 °C), protected from direct sunlight to facilitate development to L_3_ for an average of 46 days (range 15–98 days, median 42 days). Cultures were monitored microscopically for > 90% of eggs developed to third stage larvae within the egg-shell (Fig. 2). Previous work by Van Dijk & Morgan [6] observed maximal development of *N. battus* eggs to this stage in 44–52 days at temperatures between 15 and 20 °C.

### Hatching experiments

Six aliquots of approximately 500 embryonated *N. battus* eggs per population were included in the hatching experiment. Half (three aliquots) were exposed to a ‘Chill’ incubation and the other three aliquots were given a ‘non-chill’ incubation. Eggs were transferred to six well cluster plates (Sarstedt, Germany), one aliquot per well in 7 ml tap water. Each 6-well cluster plate contained two populations; three replicate aliquots per population. For the ‘Chill’ incubation, eggs were placed at 4 °C (range 2–8 °C) for 6 weeks then incubated at the optimum hatching temperature for *N. battus*; 13 °C (range 13–15 °C) for a further 4–6 weeks. Incubation temperature of 13 °C was chosen in this study based on the findings of previous work which indicated that 13 °C provided the strongest hatching stimulus for isolates from Edinburgh and Bristol [7]. For the ‘Non-chill’ incubation, eggs were incubated at 13 °C (range 13–15 °C) for 10–12 weeks without prior chilling.

Following incubation, suspensions were fixed with helminthological iodine (Lugol’s iodine). A representative 1.4ml aliquot from each well was examined and the number of third stage larvae and developed eggs were recorded.

### Data analysis

#### Binary logistic regression

Each hatching experiment was conducted in triplicate, comparison of these technical replicates was conducted using binary logistic regression analysis. Binary logistic regression was also used to compare the magnitude of hatch between UK countries during initial descriptive statistical analysis and to assess the overall impact of chilling. In all GLM analyses, the outcome response tested was whether eggs had hatched or not, each factor (temperature treatment (chill or non-chill), country or technical replicate) was included as a fixed effect, the number of days which eggs were stored in faeces at 4 °C prior to processing and the week of collection were also included as fixed effects to account for any variation in hatch rate resulting from these factors.

#### Comparison of egg hatching response with markers for benzimidazole resistance

Genotyping data for the single nucleotide mutation at codon 200 of the β-tubulin isotype 1 gene of the *N. battus* populations included in the present hatching experiment were described within a larger study by Melville et al. [11]. Briefly, thirty individual parasites from each farm population were picked at random from the population as a whole, prior to the hatching experiment and pyrosequencing was conducted as previously described [10]. Linear regression analysis was used to explore the possible correlation between the proportion of eggs hatched per isolate, with and without a period of chilling, and the F200Y resistant allele frequency of the population as a whole, obtained by pyrosequencing. Analyses were carried out using R (version 3.2.5). Statistical significance was defined at the 5% level (i.e. *P* < 0.05) for all analyses.

## Results

An average of 121 eggs/larvae were counted per replicate (range 9–412). The hatching rate of eggs was found to be comparable between technical triplicates of each hatching experiment by binomial logistic regression analysis (estimate 0.009, 95% CI: − 0.01–0.03, *P* = 0.4).

**Fig. 3:**
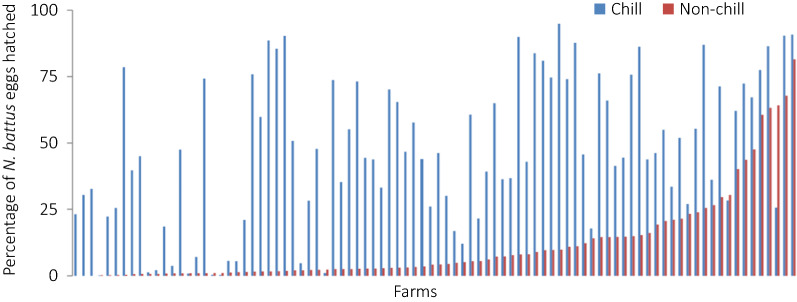
The percentage of *Nematodirus battus* eggs hatched with (blue) and without (red) a chill stimulus during *in vitro* hatching experiments from UK commercial farm populations

The proportion of the total number of eggs in suspension which hatched under experimental conditions varied greatly between farm populations (Fig. 3). The influence of the chill stimulus was assessed by binomial logistic regression analysis, and concluded that chilling increased the proportion of eggs hatched in the majority of populations, regardless of origin (OR: 0.13, 95% CI: 0.12–0.13, *P* < 2 × 10^−16^). Median hatch with and without chilling was 0.453 (range 0–0.95) and 0.0420 (range 0–0.85), respectively. Non-chill hatching was greater than chilled hatching in seven populations tested, these populations were not aggregated geographically.

Regional comparisons were conducted to assess the variability at UK level. Only two isolates were included from Wales as few samples with sufficient eggs for inclusion in the hatching experiment were collected from this region, results from these isolates were combined with those collected from England for analysis; England and Wales (*n* = 50), Scotland (*n* = 40).

**Fig. 4:**
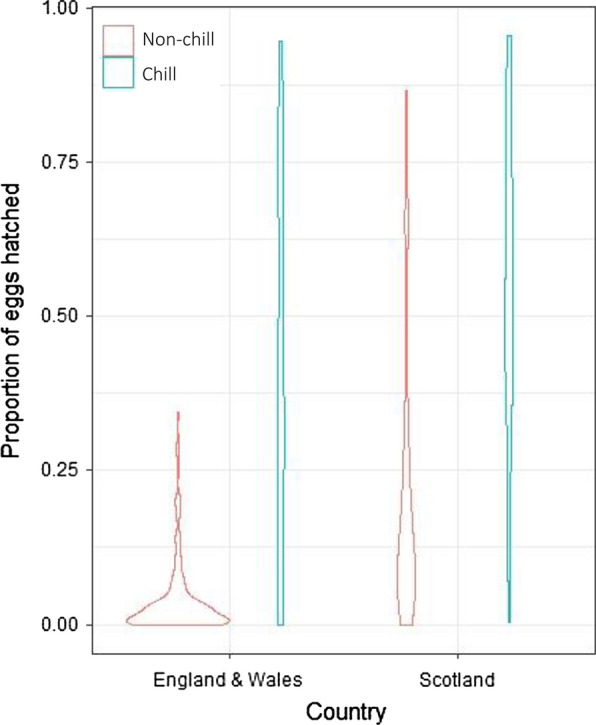
Comparison of the proportion of *Nematodirus battus* eggs hatched with (blue) and without (red) undergoing a period of chilling for populations collected from commercial farms in Scotland and those collected from England and Wales

The trend of higher hatching in Scotland was observed both with and without chilling (Fig. 4). The mean hatch rate with chilling was (mean% ± SE%) 56 ± 2.4% in Scotland (range 0–95%) and 41 ± 2.4% for England and Wales (range 0–95%). Hatching without chilling was around 5.5 times higher in Scottish isolates compared to the rest of the UK; 22 ± 2% (range 0–87%) compared to 4 ± 0.5% (range 0–35%). Binary logistic regression analysis found that the proportional hatch without prior chilling (non-chill hatching) was statistically significantly greater in populations originating from Scotland compared with those from England and Wales (OR: 2.03, 95% CI: 1.4–2.83, *P* < 0.001).

**Fig. 5:**
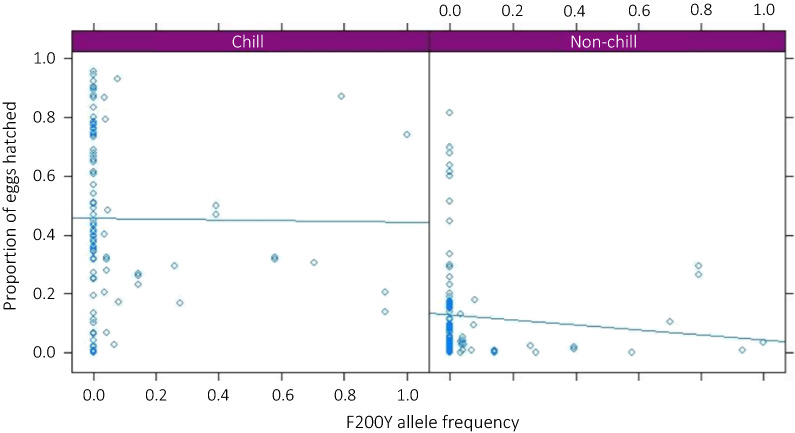
Allele frequency of the single nucleotide polymorphism F200Y of the β-tubulin isotype 1 gene, prior to the hatching experiment plotted against the proportion of *Nematodirus battus* eggs from the populations that hatched with and without a chill stimulus, with regression lines

Figure 5 shows the correlation of F200Y resistant allele frequency of the population prior to the hatching experiment and the proportion of eggs hatched with and without a chill stimulus. Regression analysis found no statistically significant associations between the constitutive resistant allele frequency of the populations and the proportion of eggs hatched with or without a chill stimulus at the 5% level (estimate − 0.008, 95% CI: − 0.16–0.15, *P* = 0.92 and estimate − 0.142, 95% CI: − 0.40–0.11, *P* = 0.27 for chill and non-chill hatching respectively) suggesting that variation in the hatching behaviour of *N. battus* is not likely to be a significant driver of the emergence of BZ-resistance in the species.

## Discussion

A sizeable proportion of *N. battus* eggs successfully hatched without a chill stimulus in the present study. Traditionally, *N. battus* eggs were believed to require a period of chilling before biologically significant levels of hatching occurred [4]. Extended exposure of eggs to low temperatures was believed to be integral to the conversion of energy reserves within larvae from lipids to sugars, increasing cold-hardiness [12] and larval longevity on pasture [7]. Given the challenge of infection and transmission in arctic conditions, increased cold-hardiness and longevity on pasture would have provided a significant advantage to the parasite however, within current UK conditions with plentiful hosts and milder winters, these adaptations may be obsolete.

The number of hatched eggs increased with the addition of a chill stimulus in the majority of populations tested, indicating that generally eggs still required a chill stimulus to hatch as previously reported [2, 4]. However, non-chill hatching was greater than chilled hatching in seven populations tested, these populations were geographically distinct and perhaps indicate that hatching without prior chilling may now be the predominant mechanism on a small proportion of farms.

Hatching in the absence of a chill has been previously documented, albeit at low levels, both historically [2, 4, 14] and more recently [6, 7, 15]. Previous studies of non-chilled hatching in *N. battus* highlighted variability between farm populations [3, 7], consistent with the present findings. Large variation in the proportion of eggs hatched after chilling observed between isolates may be due to haplotype variation between populations, i.e. genetic variability in large parasite populations within genes related to the hatching of eggs and other linked activities for example.

Hatching without chilling could increase transmission opportunities, particularly on farms where host availability is variable, e.g. given rotational grazing practices or where fields are rested over spring. Although the spring hatch still appears to be the most important for transmission of this species on the majority of farms, low level infection later in the season could safeguard the population in case the spring hatch fails. Low level infection throughout the summer would be unlikely to cause significant pathology in grazing lambs as the greatest intestinal damage is typically caused by the influx and development of large numbers of immature parasites ingested in spring [16, 17]. Education may be important particularly surrounding the interpretation of faecal egg counts to discourage whole-flock anthelmintic treatments being administered on the sight of low numbers of *N. battus* eggs during summer and autumn. Acute infection has also been observed in autumn [15], in animals not exposed to *N. battus* as lambs during spring, further complicating the advice for farmers and highlighting the need for continued knowledge exchange.

The results of the present study indicated a geographical trend; a significantly higher proportion of eggs hatched in Scottish isolates compared to those collected from England and Wales, both with and without a chill stimulus. In a previous study comparing hatching of four *N. battus* isolates, greater hatching was again observed in Scottish populations compared to those originating from England when a chill stimulus was applied however, in the absence of a chill stimulus, the opposite was reported; i.e. greater non-chill hatching in English isolates compared to those from Scotland [7]. Higher hatching without chilling in the north may appear counter-intuitive, given the reliability of cold temperatures in the north and predictions of *N. battus* spring hatch being more consistent in Scotland compared to southern regions in the face of climatic warming [18]. However, hatching is likely to be influenced by a number of environmental and management factors.

Control of *N. battus* on farm relies heavily on predicted egg hatch dates to inform monitoring and anthelmintic treatment with use of online risk maps being reported by a third of farmers surveyed (our unpublished data). Other control strategies such as avoidance grazing, where young lambs are not grazed on the same plots in consecutive years during spring, have been shown to reduce *N. battus* pasture contamination [19]. However, regional uptake and the potential selection pressure on variable hatching responses resulting from the advocated control strategies have not yet been studied. It could be hypothesised that the observed geographical trend of greater non-chill hatching in the North of the UK may reflect a variation in farm management practice rather than a parasite or environmental factor. Detailed knowledge of the expected timing of *N. battus* egg hatching, the mechanism of adaptive variation and/or the factors which influence it are therefore pertinent to inform prediction models and review current management strategies for the effective control of this parasite species.

The mechanism of adaptive variation in *N. battus* hatching remains unclear. The requirement for a chill stimulus may be under genetic control and as such, could be selected for over time. Alternatively, hatching may follow a bet-hedging approach where genotypically similar eggs are stimulated to hatch with or without chilling by factors within the host (e.g. immune factors or parasite signals) or the environment. Several environmental factors, particularly extremes (maximum and minimum) in spring temperature, have been highlighted by previous studies as potential drivers of hatching in *N. battus* eggs [6, 7].

It was hypothesised that the emergence of benzimidazole resistance may be associated with the hatching of *N. battus* eggs out-with the traditional spring hatch, suggesting that populations active throughout the grazing season may be exposed to a greater number of anthelmintic treatments. No statistically significant correlation was identified between resistant allele frequency (F200Y) and the proportion of eggs hatched with or without a chill stimulus. Non-chill hatching is therefore unlikely to be a significant driver of the emergence of BZ-resistance in the species. Variable hatching of eggs may reduce the selection pressure from treatment as not all larvae would be exposed at any one time. The lack of association between the proportion of eggs hatched after chilling and resistant allele frequency suggests that the mutation is not associated with significant fitness costs in these isolates, in agreement with BZ-resistance in other trichostrongylid species [20].

The present study examined a snap-shot from each population to explore the requirement for a chill stimulus at the individual and population levels. Close monitoring of a number of populations longitudinally, for example, using repeated hatching experiments would provide valuable information on the hatching dynamics of *N. battus* eggs over time, both within and between grazing seasons.

## Conclusions

Hatching behaviour in *N. battus* appears to be a plastic attribute, varying between populations. The results of the present study indicate that a large number of eggs are able to hatch without chilling in some populations, providing a significant larval challenge out-with the expected spring window. Further investigation of the factors influencing the requirement for chilling prior to hatching in this species would be beneficial to explore the substantial variation in egg hatching in the absence of a chill stimulus observed in the present study. Greater sampling within regions to explore the fine scale variation between individual farms and how this is influenced by management practices would also be valuable, informing the development of novel, sustainable on-farm control strategies.

## Data Availability

The datasets generated during the present study are not publicly available due to ongoing analysis of the dataset within other aspects of the project but may be available from the corresponding author on reasonable request.

## References

[1] Wright N. Vickers M. ADAS: Economic impact of health and welfare issues in beef cattle and sheep in England; 2013. http://beefandlamb.ahdb.org.uk/wp-content/uploads/2013/04/Economic-Impact-of-Health-Welfare-Final-Rpt-170413.pdf. Accessed 16 Jul 2020.

[2] Boag B, Thomas RJ (1975). Epidemiological studies on *Nematodirus* species in sheep. Res Vet Sci..

[3] Thomas DR (1990). The epidemiology of *Nematodirus battus*—is it changing?. Parasitology..

[4] Thomas RJ, Stevens AJ (1960). Ecological studies on the development of the pasture stages of *Nematodirus battus* and *Nematodirus filicollis*, nematode parasites of sheep. Parasitology..

[5] Hoberg EP (2005). Coevolution and biogeography among Nematodirinae (Nematoda: Trichostrongylina) Lagomorpha and Artiodactyla (Mammalia): exploring determinants of history and structure for the northern fauna across the Holarctic. J Parasitol..

[6] Van Dijk J, Morgan ER (2008). The influence of temperature on the development, hatching and survival of *Nematodirus battus* larvae. Parasitology..

[7] Van Dijk J, Morgan ER (2010). Variation in the hatching behaviour of *Nematodirus battus*: polymorphic bet hedging?. Int J Parasitol..

[8] Lacey E, Gill JH (1994). Biochemistry of benzimidazole resistance. Acta Trop..

[9] Mitchell S, Mearns R, Richards I, Donnan AA, Bartley DJ (2011). Benzimidazole resistance in *Nematodirus battus*. Vet Rec..

[10] Morrison AA, Mitchell S, Mearns R, Richards I, Matthews JB, Bartley DJ (2014). Phenotypic and genotypic analysis of benzimidazole resistance in the ovine parasite *Nematodirus battus*. Vet Res..

[11] Melville LA, Redman E, Morrison AA, Chen PCR, Avramenko R, Mitchell S (2020). Large scale screening for benzimidazole resistance mutations in *Nematodirus battus*, using both pyrosequence genotyping and deep amplicon sequenicng, indicates the early emergence of resistance on UK sheep farms. Int J Parasitol Drug Drug Resist..

[12] Ash CPJ, Atkinson HJ (1983). Evidence for a Temperature-dependent conversion of lipid reserves to carbohydrate in quiescent eggs of the nematode, *Nematodirus battus*. Comp Biochem Phys..

[13] Jackson F, Christie M (1972). Quantitative recovery of floatable helminth eggs from 1g of ruminant faeces for counting followed by hatching for identification. Trans R Soc Trop Med Hyg..

[14] Gibson TE, Everett G (1981). Ecology of the free living stages of *Nematodirus battus*. Res Vet Sci..

[15] Sargison ND, Wilson DJ, Scott PR (2012). Observations on the epidemiology of autumn nematodirosis in weaned lambs in a Scottish sheep flock. Vet Rec..

[16] Kates KC, Turner JH (1955). Observations on the life cycle of *Nematodirus spathiger*, a nematode parasitic in the intestine of sheep and other ruminants. Am J Vet Res..

[17] Mapes CJ, Coop RL (1972). The development of single infections of *Nematodirus battus* in lambs. Parasitology..

[18] Gethings OJ, Rose H, Mitchell S, Van Dijk J, Morgan ER (2015). Asynchrony in host and parasite phenology may decrease disease risk in livestock under climate warming: *Nematodirus battus* in lambs as a case study. Parasitology..

[19] Black WJM (1959). A grassland management method of controlling *Nematodirus* infestation. J British Grassland Soc..

[20] Elard L, Sauve C, Humbert JF (1998). Fitness of benzimidazole-resistant and -susceptible worms of *Teladorsagia circumcincta*, a nematode parasite of small ruminants. Parasitology..

